# Genomic and epidemiological characteristics of *Shigella boydii* in Australia, 1991–2022

**DOI:** 10.1080/20477724.2025.2573308

**Published:** 2025-10-23

**Authors:** Aaliya F Ibrahim, Danielle J Ingle, Jessica R Webb, Benjamin P Howden, Patiyan Andersson, Benjamin G Polkinghorne, Rose Wright, Kathryn Glass, Martyn D Kirk

**Affiliations:** a National Centre for Epidemiology and Population Health, Australian National University, Canberra, Australia; b Interim Australian Centre for Disease Control, Australian Government Department of Health and Aged Care, Canberra, Australia; c Department of Microbiology and Immunology, The University of Melbourne at the Peter Doherty Institute for Infection and Immunity, Melbourne, Australia; d Centre for Pathogen Genomics, The University of Melbourne, Melbourne, Australia; e School of Biological Sciences, The University of Adelaide, Adelaide, Australia; f Microbiological Diagnostic Unit Public Health Laboratory, The Department of Microbiology and Immunology, The University of Melbourne at the Peter Doherty Institute for Infection and Immunity, Melbourne, Australia

**Keywords:** Shigella, Shigellosis, Genomics, Infectious Diseases Epidemiology, Antimicrobial Resistance

## Abstract

Shigellosis is a leading cause of diarrheal mortality worldwide. *Shigella boydii* is one of four *Shigella* species that contributes to this burden, however studies on *S. boydii* are limited. Here we combined epidemiological and genomic data to better understand *S. boydii* circulating both in Australia and globally. Between 1991 and 2019, there were 294 cases of *S. boydii* infections notified to the National Notifiable Diseases Surveillance System by Australian states and territories, with an increasing trend in notifications observed from 2013. Of cases whose place of acquisition was known, 54% (111/206) were acquired overseas, mainly from South-East Asia (57%; 63/111). Our genomic analysis included 250 *S. boydii* isolates: 44 from Victoria, Australia spanning 22 years (2001–2022) and 206 international isolates spanning 91 years (1930–2020). Phylogenomic analyses identified five major *S. boydii* phylogenetic lineages circulating globally. The Australian isolates were distributed across all five lineages, but the highest proportion was in Lineage 3. Antimicrobial resistance was common in both international and Australian isolates with > 60% of isolates classified as multi-drug-resistant. Resistance to the main clinically relevant antimicrobials was rare in *S. boydii*. Ciprofloxacin resistance was detected in seven *S. boydii*, however reduced susceptibility to ciprofloxacin was detected in 56 isolates and found in both Australian and international data. Importantly, resistance mechanisms to third-generation cephalosporins and macrolides were also detected. This study is the largest genomic analysis of *S. boydii* to date, providing insights into the population structure, epidemiology and emerging AMR threats in this neglected *Shigella* species.

## Introduction

Shigellosis is a diarrheal disease caused by the gram-negative bacteria *Shigella spp*. Transmission primarily occurs via the fecal oral route, through direct or indirect contact with fecal matter [1]. In low- and middle-income countries (LMICs), the burden of shigellosis is greatest in young children, while in high-income countries, incidence is typically highest in returning travelers and in men who have sex with men (MSM) [2]. In Australia, most outbreaks of shigellosis have been associated with person-to-person transmission rather than food or water-borne outbreaks [3]. Globally, *Shigella* was the fourth highest cause of global diarrheal mortality in 2021, resulting in 117,000 deaths (95% UI. 71,900–178,000) [4]. *Shigella boydii* is one of four *Shigella* species, with *Shigella sonnei* and *Shigella flexneri* being leading causative agents for the global burden of shigellosis. The prevalence of *S. boydii* is highest in the Indian subcontinent [5]. In Australia, *S. boydii* accounted for less than 1% of approximately 18,000 shigellosis cases notified between 2001 and 2019 [6].

While shigellosis is usually a self-limiting disease, treatment with antibiotics may be recommended for certain high-risk groups to reduce the severity and duration of illness [7]. While treatment guidelines vary internationally, ciprofloxacin (in the fluoroquinolone class of antibiotics) is generally recommended as the first-line treatment for shigellosis, including in Australia [8]. Recommended second-line therapies include azithromycin (macrolide class of antibiotics), ceftriaxone (third-generation cephalosporins (3GCs)) and trimethoprim-sulfamethoxazole [9]. In recent years, treatment of shigellosis has become complicated with the increasing prevalence of broad-spectrum antimicrobial resistance (AMR), which has been reported among all *Shigella* species, including *S. boydii* [10].

Genomic epidemiology incorporating whole genome sequencing (WGS) and public health surveillance data has transformed our understanding of enteric bacterial pathogens globally. Traditionally, the classification of *Shigella* into the four species and into > 50 serotypes was based on their O-antigen type and biochemical properties [6]. However, these conventional methods are laborious, time-consuming and difficult to automate [11]. Genomic epidemiological analyses now leverage routinely produced WGS data to characterize bacterial genomes, providing greater resolution than historical approaches. Standard methods include multilocus sequence typing (MLST), explorations of the evolution of populations through phylogenomics and characterization of known mechanisms of AMR [12,13]. Genomic analysis has shown that *Shigella* species are paraphyletic members of the *Escherichia coli* species, which have converged via parallel evolutionary processes through the acquisition of mobile elements and loss of gene function [14]. Studies examining the population structure of *S. boydii* are limited. Currently available data indicates that *S. boydii* do not share a common ancestor and are broadly distributed across multiple clades in the *E. coli* phylogeny [15]. An earlier study using a small dataset of 72 isolate genomes identified three phylogenomic clades for *S. boydii* [16]; however, this study did not include any isolates from the Americas, Europe or Australia. Further, the prevalence of AMR determinants has not been widely reported to date in *S. boydii*, with existing studies primarily focussing on data from LMICs only [17].

The development of fluoroquinolone resistance in *Shigella* can be associated with multiple mechanisms, including acquisition of fluoroquinolone-resistant genes or mutations in the quinolone resistance determining region (QRDR) of the chromosomal genes encoding DNA gyrase (*gyrA* and *gyrB*) and topoisomerase IV (*parC* and *parE*) [18]. Triple mutations in the QRDR have been reported to confer resistance to ciprofloxacin, while single and double mutations confer reduced susceptibility [19]. Other important resistance markers in *Shigella* species include extended-spectrum β-lactamase (ESBL) genes, such as those in the bla_CTX-M_ family, which confers resistance to extended-spectrum cephalosporins (e.g. ceftriaxone); *mph(A)*, which confers resistance to azithromycin; and *dfrA1* and *sul2* (trimethoprim-sulfamethoxazole resistance) [20].

There is limited published evidence on the epidemiology, genomic characteristics, genotypic AMR trends and population-level structure of *S. boydii*. In this study we analyzed Australian notification data from 1991 to 2019 to elucidate epidemiological trends and risk factors including, age, sex and travel history. We also explored the population structure, geographical clustering and AMR prevalence in *S. boydii* circulating globally for 93 years (local Australian dataset: 44 isolates and international dataset: 206 isolates). This study is the largest genomic analysis of *S. boydii* to date and provides insights into the population structure, epidemiology and emerging AMR threats in this neglected *Shigella* species.

## Methods

### Epidemiological analysis

We analyzed notifications of *S. boydii* reported to Australia’s National Notifiable Diseases Surveillance System (NNDSS: https://nindss.health.gov.au/pbi-dashboard/) from 1991 to 2019. In Australia, doctors and laboratories are required to report all cases of shigellosis to one of eight state or territory health departments, depending on the state of diagnosis. States and territories then report cases to the NNDSS. Data from New South Wales was only available from 2001 when shigellosis became notifiable in that state.

We collected de-identified data on all cases of *S. boydii* reported to the NNDSS from 1991 to 2019, including the following variables: sex, 5-year age group, state, month and year of diagnosis and country where the infection was likely to have been acquired. NNDSS data were provided by the Department of Health and Aged Care, on behalf of the Communicable Diseases Network Australia.

The country of acquisition for overseas acquired cases were grouped in accordance with World Health Organization regions: South-East Asian Region, Western Pacific Region, Eastern Mediterranean Region, African Region, Americas Region and European Region.

We calculated notification rates per 100,000 population using mid-year residential population estimates from the Australian Bureau of Statistics for the years 1991 to 2019. We used a negative binomial regression model to evaluate changes in notification rates over time, using year, post-2013 and pre-2001 as predictors. Year was modeled as a continuous variable to evaluate overall temporal trends, while the pre-2001 period was included as a binary predictor to adjust for the changes in population structure from 2001 (due to the inclusion of NSW data). Additionally, noting the observed increasing trend in notifications from 2013, we included an interaction term between year and a binary indicator (post-2013) in the model to assess changes in trends during this period. Results are presented as notification rate ratios (NRR) and statistical significance was defined as a p-value of less than 0.05. StataCorp StataMP 17 was used for analysis and creation of graphs.

### Curation of the short read whole-genome sequencing dataset

The Microbiological Diagnostic Unit Public Health Laboratory (MDU PHL) at the University of Melbourne is the bacterial reference laboratory for the state of Victoria. For our genomic analysis, we collected all *S. boydii* isolates referred to MDU PHL between 2001 and 2022 that had viable cultures (*n* = 47). We curated the associated metadata for the available sequences, including year and month of collection, age-group (in 5-year intervals), sex and reported international travel history. DNA extraction and whole genome sequencing (WGS) on Illumina platforms were conducted at MDU PHL, as previously described [21]. Sequencing data are available at the National Center for Biotechnology Information Short Read Archive (BioProject PRJNA857526).

For the international isolates, we reviewed the literature on *S. boydii* and included *S. boydii* genomes where short read data were publicly available, drawn from previously published WGS-based studies of *Shigella* [12,16,17,22,23]. Our sampling strategy for published data was to include isolates of *S. boydii* where short-read data conducted on the Illumina platform was available, in order to provide a comprehensive dataset of *S. boydii*. In total, data from 241 published isolates were collected.

The Illumina reads were included in the genomic analyses based on four quality control (QC) parameters. These were i) Q score ≥30, ii) minimum estimated average genome coverage of ≥30, iii) N50 > 1,000, and iv) taxonomic assignment identification as *S. boydii* by Kraken [24]. Using these criteria, 38 isolates were excluded from the study, including three Australian isolates and 35 publicly available international isolates. Details of the isolates included in the study are available in Supplementary Table S1.

### Phylogenetic analysis

All 250 isolates that passed QC were aligned to the publicly available *S. boydii* reference genome (NCBI reference number: NC_010658.1) using Snippy v4.5.5 (https://github.com/tseemann/snippy) to identify single nucleotide polymorphisms (SNPs) with default parameters. Phage regions identified using PHASTER [25] were masked from the alignment. Recombinant regions identified using Gubbins v2.4.1, with the filter percentage set to 30 [26], were also masked in the alignment, and the final core SNP alignment of 24,969 bases generated with SNP sites [27]. A maximum-likelihood (ML) phylogeny was inferred using IQ-Tree (v1.6.12) [28] using a general time reversible (GTR) substitution model +Γ, inclusive of invariable constant sites and 1,000 bootstraps. The final phylogenetic tree was midpoint rooted. The population structure and phylogenetic lineages were investigated using a hierarchical Bayesian Analysis of Population Structure (BAPS) based on the core genome SNP alignment with default parameters [29]. Pairwise SNP-distances for all isolates were determined using the final core SNP alignment and snp-dists v0.8.2 (https://github.com/tseemann/snp-dists). The ML phylogeny was visualized in R (v 4.4.1) with ggtree (v 3.14.0). ChatGPT-4o was used to assist in R coding, including troubleshooting errors in the code.

### Genome assemblies and detection of antimicrobial resistance determinants

*De novo* assemblies were performed using SPAdes (v 3.14.1) using the ‘−isolate’ flag [30]. The genome assemblies were screened for known AMR determinants using the AMRFinder [31] database (https://github.com/ncbi/amr/wiki/AMRFinder-database) as implemented in abriTAMR tool [13] (https://github.com/MDU-PHL/abritamr) with the species flag of ‘*Escherichia’*.

### Antimicrobial resistance profiles

Isolates were classified as multidrug resistant (MDR) when genetic mechanisms pertaining to three or more of the antimicrobial classes – i.e. β-lactams, macrolides, fluoroquinolones, aminoglycosides, sulfonamides, tetracyclines and trimethoprim – were detected, and as extensively drug-resistant (XDR) when resistance markers for more than five of the antimicrobial classes were identified. Genetic resistance markers that did not fit into one of the seven classes mentioned above, were grouped into an ‘other’ category, which was excluded from the calculation for MDR or XDR. Further, *AmpC* β-lactamases (annotated as *blaEC*) were intrinsic in all isolates [32]. Therefore, presence of these genes was not included in the calculations for MDR or XDR. Isolates were considered resistant to ciprofloxacin if triple mutations were detected in the quinolone resistance determining regions (QRDRs) of *gyrA, gyrB, parC* or *parE*, or if a single point mutation and a *qnr* gene was detected. Isolates with single or double point mutations [19], or a *qnr* gene alone were inferred to have reduced susceptibility to ciprofloxacin.

### Ethics statement

We obtained ethical approval for this study from the Australian National University Human Research Ethics Committee [protocols: 2018/560 and 2021/038] and the ACT Health Human Research Ethics Committee’s Low Risk Sub-Committee [2018/ETH/00158]. Data were collected in accordance with the Victorian Public Health and Wellbeing Act 2008. Ethical approval was also received from the University of Melbourne Human Research Ethics Committee (study number 1,954,615.3). No participants were recruited for this study, and all data used were previously collected during public health investigations. Given that de-identified data was collected, representing infections occurring several years ago, it was not feasible to seek individual consent, nor to inform participants about the use of data in this study.

## Results

### National epidemiology of S. boydii

**Figure 1.**
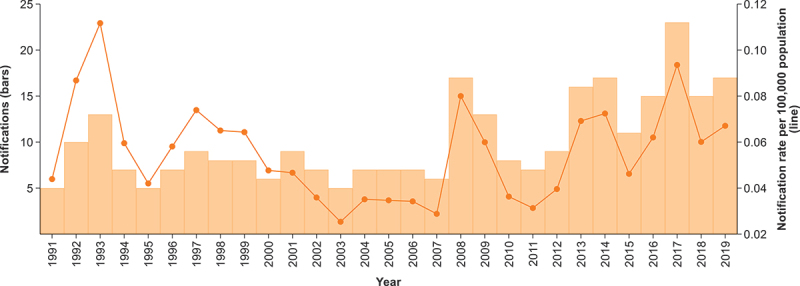
Notifications and crude notification rate of *S.* *boydii*, Australia, 1991–2019.

Between 1991 and 2019, there were 294 cases of *S. boydii* notified nationally in Australia, representing 1.2% (294/25,159) of all shigellosis cases reported over that period. While rates of *S. boydii* infection remained low nationally (< 1 case per 100,0000 population), there was an increasing trend in notification rates from 2013 (yearly NRR = 1.0002; *p* = 0.046) (Figure 1). Over the 29-year period, while Victoria was the jurisdiction with the highest number of reported cases (*n* = 111), notification rates were highest in Northern Territory (2.8 cases per 100,000 population), followed by South Australia (2.6 cases per 100,000 population) (Table S2).

*The bars and left y-axis show number of notifications; lines and right y-axis show notification rates.*

**Figure 2.**
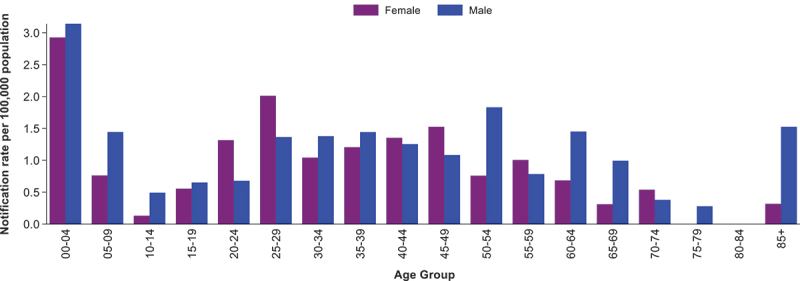
Notification rate of *S.* *boydii* by age group and sex, Australia, 1991–2019.

The highest notification rates were observed in those aged less than 5 years, followed by those aged 25 to 29 years (Figure 2). Notification rates were higher in males across most age groups, except in those aged 20–29, 40–49, 55–59 and 70–74 years, where rates were higher among females.

*Excludes cases where age and/or sex was unknown (n=9).*

From 2006 there was an increase in available data on place of acquisition for *S. boydii* cases in NNDSS, with place of acquisition known for 82% (148/181) of cases; prior to this (1991–2005), place of acquisition was recorded only for 51% (58/113) of cases and of those, the majority (97%; 56/58) were reported to be acquired in Australia (Figure S1). Overall, cases from 1991 to 2019, for which place of acquisition was known, 54% (111/206) were acquired overseas. The most common region of acquisition was the South-East Asia region (56.8%; 63/111), followed by the Eastern Mediterranean (14.4%; 16/111) and African (11.7%; 13/111) regions (Table 1). The most common acquisition countries were India and Indonesia, accounting for 30% (33/111) and 19% (21/111) of cases acquired overseas, respectively.

**Table 1.** Overseas acquired *S. boydii* notifications by region of acquisition, Australia, 1991–2019.

Region of Acquisition	Cases	Proportion (%)
South-East Asia	63	56.8
Western Pacific	6	5.4
Eastern Mediterranean	16	14.4
Africa	13	11.7
Americas	5	4.5
Europe	2	1.8
Overseas - region unknown	6	5.4
Total	111	100

### Global population structure of S. boydii

**Figure 3.**
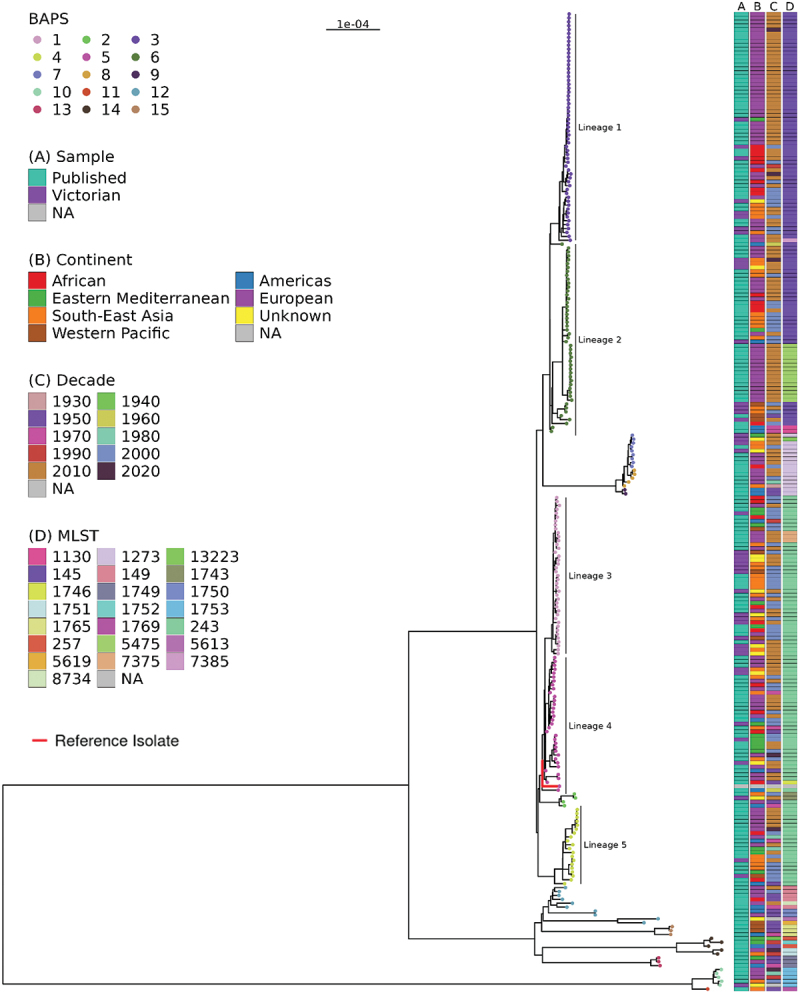
Maximum-likelihood core genome phylogeny of 250 *S. boydii* genomes and the reference genome.

We analyzed the genomes of 250 isolates of *S. boydii* extracted from human clinical samples, including 44 isolates from Victoria and 206 published international isolates. The published genomes represented isolates from 27 countries, six regions and spanning nearly a century (1930 to 2020) (Figure S2). Most of these isolates were from France (52.9%; 109/206), followed by Bangladesh (7.3%; 15/206) and the United States (6.8%; 14/206). No clear geographical clustering within the tree was observed, although more than half of the isolates were from Europe. The Australian isolates were distributed across the ML phylogeny (Figure 3). Five major lineages were identified, L1-L5, defined by ≥ 20 isolates in the BAPS clusters (BAPS groups 3, 6, 1, 5 and 4), with an additional 10 BAPS clusters identified.

ML tree of 250 *S.* *boydii* genomes included in this study, with 44 isolates from Victoria, Australia and 206 isolates from published literature. BAPS groups are highlighted in the phylogenetic tree tip points, with five corresponding lineages identified (L1-L5). The reference genome (in L4) is represented with a red branch colour. For each isolate, the following associated data are shown: A) origin of sample; B) continent of sample collection for published isolates and travel history for Victorian isolates; C) decade of sample collection; and D) MLST type.

Isolates in lineages L1-L5 represented 81.2% (203/250) of all isolates included in the study, with most isolates in L1 (*n* = 59), followed by L2 (*n* = 49), L3 (*n* = 41), L4 (*n* = 34) and L5 (*n* = 20). L1, which consisted of isolates collected from 1994 to 2020, comprised mostly international isolates from the European region, predominantly from France. In contrast, the Australian L1 isolates (*n* = 8) had a range of travel histories, including from the African, Eastern Mediterranean, South-Asian and European regions. Similarly, L2 had a temporal span of 69 years (1954–2022), and most isolates were from the European region; however, there were subgroups of isolates from the South-East Asia region. L3 comprised 41 isolates with a temporal span of 21 years. L3 had the highest number of Victorian isolates (*n* = 15), including an eight-case cluster (20 pairwise SNP threshold), which had isolate collection dates ranging from June 2002 to September 2019. Australian case travel histories within this cluster varied, with three cases having a history of travel to India, one case a history of travel to Zimbabwe, two cases who did not travel and two cases for which the travel history was unknown. When looking at overall temporal trends, L1-L3 had the highest proportion of recent isolates (those collected after 2000); L4 had a temporal span of 71 years (1950–2020) and L5 spanned 65 years (1956–2020). Further, the major lineages broadly aligned with MLST groupings, with L1 congruent to ST145, L2 split between ST145 and ST5475 (which are single-locus variants) and L3-L5 predominantly aligned to ST243. In total, 22 STs were detected with ST congruent with the ML phylogeny and BAPs clusters (Table S1). There were 42 isolates included in this study that were common to the previous largest genomic analysis of *S. boydii*, which defined three phylogenomic clades (Clades 1–3) for the species [16]. Of these, there were 21 isolates from Clade 3, which primarily corresponded to L1 and L2 in this study; 20 isolates from Clade 2, corresponding to lineages L3-L5; and one isolate from Clade 1, which did not correspond to any of the major lineages (Figure S3).

## Antimicrobial resistance patterns in S. boydii

**Figure 4.**
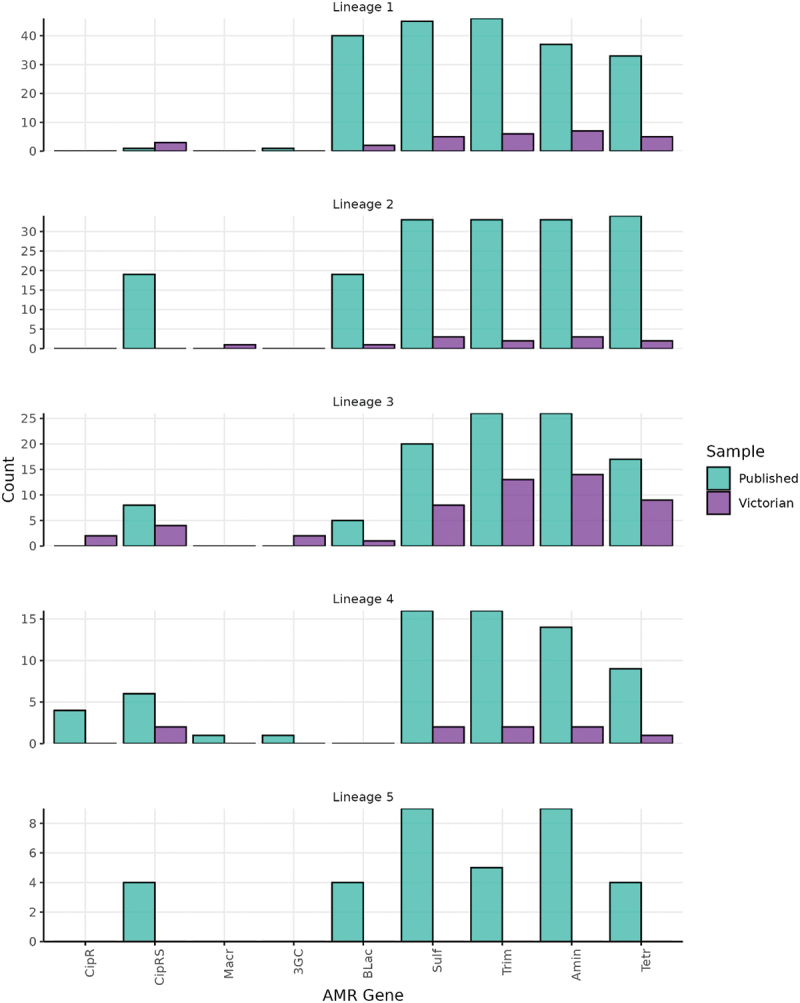
Prevalence of selected AMR genes in *S.* *boydii* genomes, by phylogenetic lineage.

There were high levels of AMR determinants present in both international and Australian isolates, with > 60% (168/250) of isolates found to be MDR (Table 2). The most common resistance mechanism detected was trimethoprim, which was detected in 66.8% (167/250) of isolates, followed by aminoglycosides (66.4%; 166/250) and sulfonamides (64.8%; 162/250). There were high levels of co-occurrence of *sul* and *dfr* genes, suggesting co-trimoxozole resistance. While determinants for fluoroquinolone resistance were present in 22.3% (46/206) and 38.6% (17/44) of international and Australian isolates, respectively, only seven isolates harbored genes conferring resistance to ciprofloxacin. Four of these ciprofloxacin-resistant isolates were international isolates collected from France and identified in L4, and the remaining isolates were from Victoria: two in L3 and one not in a major lineage, with a travel history to India (Figure 4). Four isolates contained AMR genes that confer resistance to ceftriaxone which is a 3GC; two were Victorian isolates with unknown travel histories in L3 with *bla*_*CTX-M-15*_, and two were international isolates collected in France, with one from L1 that carried a *bla*_*CTX-M-3*_, and one from L4 that carried *bla*_*DHA-1*_ (an *AmpC* mechanism for 3GC resistance). Finally, two isolates had *mphA* resistance genes detected: one international isolate in L4 collected from France and one Victorian isolate from L2 that had a travel history to Cambodia (Table S1 and Figure S4).

Note, the y-axis is not consistent for all lineages. Abbreviations: Quinolone (Quin), Ciprofloxacin resistant (CipR), Ciprofloxacin reduced susceptibility (CipRS), Macrolide, 3GC, Beta lactamase: not-ESBL or carbapenemase (BLac), Sulfonamide (Sulf), Trimethoprim (Trim), Aminoglycoside (Amin), Tetracycline (Tetr). Isolates that are not in L1-L5 are not included in the graph (n=47).

**Table 2.** Prevalence of selected *S. boydii* antimicrobial resistance genes in Victoria, Australia and internationally.

Antibiotic class	Genetic markers#	Prevalence	
		International (*n*=206)	Victorian (*n*=44)
**Beta-lactams**		82 (39.8%)	11 (25.0%)
Third-generation cephalasporins	blaCTX-M-15| blaCTX-M-3|blaDHA-1	2 (1.0%)	2 (4.5%)
Non-ESBL or carbapenemase	blaOXA-1| blaOXA-1*| blaOXA-1*^| blaTEM-1| blaLAP-2;blaTEM-1	80 (38.8%)	9 (20.5%)
**Macrolides**	mph(A)	1 (0.5%)	1 (2.3%)
**Fluoroquinolone**		46 (22.3%)	17 (38.6%)
Ciprofloxacin resistant	gyrA_D87N;gyrA_S83L;parC_S80I |gyrA_D87Y;qnrS1|gyrA_S83L;qnrS1|gyrA_S83L;qnrB4	4 (1.9%)	3 (6.8%)
Ciprofloxacin reduced susceptibility	gyrA_D87Y|gyrA_S83L|gyrA_D87G|qnrB19|qnrS1|qnrS13	42 (20.4%)	14 (31.8%)
**Sulfonamides**	sul1|sul1*|sul1*;sul2|sul1;sul2|sul2|sul2*^|sul2;sul1*^|sul3	140 (68.0%)	22 (50.0%)
**Tetracyclines**	tet(A)|tet(A)*|tet(A);tet(B)|tet(B)	114 (55.3%)	22 (50.0%)
**Trimethoprim**	dfrA1|dfrA1;dfrA14|dfrA1;dfrA5|dfrA12|dfrA14|dfrA17|dfrA7	139 (67.5%)	28 (63.6%)
**Aminoglycosides**	aadA1|aadA1*|aadA1*^|aadA1;aph(3’’)-Ib*;aph(6)-Id|aadA1;aph(3’’)-Ib;aph(6)-Id|aadA1;aph(3’’)-Ib;aph(6)-Id*|aadA1;aph(6)-Id*^|aadA1;aph(6)-Id;aph(3’’)-Ib*^|aadA2|aph(3’’)-Ib;aph(6)-Id|aph(3’’)-Ib;aph(6)-Id*^|aph(3’’)-Ib;aph(6)-Id;aadA1*^|aph(6)-Id;aph(3’’)-Ib*^	135 (65.5%)	31 (70.5%)
**MDR**		138 (67.0%)	30 (68.1%)
**XDR**		21 (10.2%)	3 (6.8%)

*Close match - when 90 to <100% of a protein in the AMRfinder database is covered by a contig at 90 to <100% identity.

^Partial match − 50% to <90% of a protein in the AMRfinder database is covered by a contig at >90% identity.

## Discussion

In this global study of *S. boydii*, we describe long-term epidemiological trends in Australia and the genomic characteristics of strains in international databases. Nationally, we observed an increasing trend in *S. boydii* notifications since 2013. This may be in part due to changing testing practices, with an increasing use of culture independent diagnostic testing (CIDT) for shigellosis in Australia since 2014 [6]. CIDT is more sensitive than culture-dependent methods and may also lead to increased reflex culture for isolate confirmation, resulting in higher notification rates. Consistent with other *Shigella* species, notification rates of *S. boydii* in Australia were highest in those aged 0–4 years old [6]. Young children are at highest risk of shigellosis due to poorer hygiene. In LMICs, transmission in this cohort may predominantly occur from contaminated food and water and poor sanitation; in high-income countries, transmission often occurs between children in childcare settings through close contact, inadequate handwashing after nappy-changing or fecal contamination of play areas [33,34].

Where place of acquisition was known, the majority of *S. boydii* notifications in Australia were acquired overseas. The most common region of acquisition was the South-East Asia region, and the most frequent country of acquisition was India. This is consistent with the global epidemiology of *S. boydii*, where the species is largely endemic to the Indian subcontinent [35]. In India, there is temporal and geographical variation in the prevalence of *S. boydii*, with various studies in different regions showing *S. boydii* prevalence among all *Shigella* isolates ranging from 2.3% to 15.0% [36–38]. Expanding to neighboring countries, in a large multicentric study of *Shigella* in China, Thailand, Indonesia, Vietnam, Pakistan and Bangladesh from 2000 to 2004, *S. boydii* was isolated from 6% of shigellosis cases overall, with the highest detection in Bangladesh (23%), followed by Pakistan (11%) [39]. While there is geographical variation in the prevalence of *S. boydii* within the Indian subcontinent, the overall occurrence relative to other species is higher than that detected in Australia, where *S. boydii* accounted for 1% of notified cases of shigellosis from 1991 to 2019.

Genomic analysis identified five major phylogenetic lineages for *S. boydii* across the global and Australian datasets. While Australian isolates were dispersed throughout all five lineages, there was a greater predominance in L3, which was characterized by higher geographical diversity than other lineages. There were no persisting clusters of Australian isolates, which suggests there is ongoing importation of *S. boydii* into Australia associated with travel, which aligns with the national notification data that shows a high proportion of cases acquired their infection overseas. It is important to note that the lineages identified in this study do not necessarily share a common ancestor. As highlighted in previous studies, *S. boydii* are not monophyletic and are instead spread across the *E. coli/Shigella* phylogeny [14]. As the primary focus of this study was epidemiological in nature, rather than a detailed investigation into the evolutionary spread of the species, we only studied isolates of *S. boydii*. To further contextualize the genomic population structure of *S. Boydii*, future genomic focussed studies should include analysis of *S. boydii* alongside other *Shigella* species and *E. coli*. To enable backward compatibility, in our study we annotated isolates using clade names designated in a previous genomic study of Global Enteric Multicentre Study (GEMS) data. The GEMS study, which analyzed genomic diversity of *E. coli* and *Shigella* isolates predominantly from sub-Saharan Africa and South Asia [40], identified three phylogenomic clades for *S. boydii* [16]. In our analysis, we included several GEMS isolates showing that the population structure of the previous study broadly matched our study, with lineages L3-L5 corresponding to the previously defined Clade 2, and L1 and L2 corresponding to Clade 3. As we included a greater number of isolates in our study, with more temporal and geographic dispersion, we were able to identify clusters with more granularity than those previously identified. Further, our genomic analysis showed that while MLST are generally reliable indicators to determine high-level groupings and population structures, the data provided through WGS and SNPs identify more granular clusters that can support more detailed surveillance and epidemiological investigations. As genomic sequencing becomes more affordable and widely used, the application of more advanced typing schemes should be considered to enable robust surveillance of shigellosis.

In our study, we found a high prevalence of AMR determinants with 168 (> 60%) isolates classed as MDR, which were most common in lineages L1-L3. Further, our study showed AMR was emerging to clinically relevant therapeutics. Over one third of Victorian isolates had at least one point mutation in QRDR compared to 9% of international isolates. Single and double mutations in the QRDR confers reduced susceptibility to ciprofloxacin, and are of particular concern as they have been shown to be evolutionary intermediates on the path to acquiring full resistance [33]. The prevalence of QRDR mutations detected in our study (13.2%) is consistent with other studies. For example, a study of WGS data of *Shigella* collected in LMICs from 2007 to 2011 found that QRDR point mutations were present in 15% of *S. boydii* isolates, which was much lower than observed in all other *Shigella* species [17]. Notably in our study, while resistance mechanisms to 3GC and macrolides were detected in the *Shigella* data, these were at low frequency. The isolates carrying these genes were dispersed across lineages and among both international and Australian isolates, suggesting that population expansion of these AMR determinants in *S. boydii*, e.g. via an AMR plasmid, is not yet occurring. This contrasts with the recent reports of plasmid-driven MDR in both *S. sonnei* and *S. boydii* [20,32,41]. Detailed exploration of the *bla*_*CTX-M−15*_, *bla*_*CTX-M−3*_, *bla*_*DHA-1*_ and *mphA* genes, for example if they are carried on plasmids, was beyond the scope of this study, however a future avenue of research would be to undertake long read sequencing on ONT platforms to investigate the locations and arrangements of the AMR genes detected in this study. There was high prevalence of AMR to several second-line treatments in our study, including beta-lactams (non-ESBL or carbapenemase), and sulfonamides and trimethoprim (*sul* and *dfr* genes). Broadly, our results are consistent with published trends, which highlight a global rise in broad-spectrum AMR in *Shigella* species in recent decades [42].

The prevalence and diversity of AMR mechanisms, including to 3GCs, highlights the urgent need for the continued development of novel treatments and interventions, including vaccines and monoclonal antibodies, particularly in LMICs [7]. In high-income countries, our results emphasize the need to increase awareness among clinicians that MDR in *S. boydii* is relatively common, to inform clinical decision making. A positive finding in our study was the low levels of macrolide resistance markers, indicating that treatment of *S. boydii* infection with azithromycin, which is often recommended in regions that have high rates of resistance to ciprofloxacin, may be appropriate [42]. These data can be used to provide evidence in the development of treatment guidelines and policy for shigellosis management.

There were some limitations associated with our study. Firstly, it is likely that there was an underestimation of overseas acquired cases based on the NNDSS dataset. Prior to 2006, there were only two cases whose place of acquisition was noted to be outside of Australia and these were classified as ‘Overseas-country unknown,’ with no further information on the country of acquisition. From 2006, there was an increase in reporting place of acquisition information, including detailed data on country of acquisition. This trend appears to indicate an expansion of the NNDSS data specifications to include country of acquisition from 2006 onwards.

We had limited metadata associated with the genomic sequences from Victoria, such as Indigenous status and sexual history. There are several known high-risk populations for shigellosis in Australia, including First Nations people and men who have sex with men [6]. The only metadata we had in our study for Victorian strains were international travel history and age-group, which minimized our ability to assess risk factors beyond travel history and age. Metadata was also limited for international isolates, with year and country of sample collection the only data that was available consistently across all studies.

We did not have genomic data on *S. boydii* from other Australian states and territories. Noting the distinct differences in the demography of Australian jurisdictions, genomic analysis of isolates from a single state may not be generalizable to the entire Australian population. Further, while we included all Victorian isolates from 2001 to 2022 available for sequencing, this represents only a portion of notified Victorian cases over that period. Increasing use of culture-independent testing for enteric pathogens limits the availability of isolates, thereby compromising the ability to undertake further analysis using WGS [43]. Noting the enhanced data that WGS can provide for the characterization of *Shigella* and other enteric pathogens, the least of which is the detection of resistance markers, it is imperative that culturing of isolates continue until metagenomic approaches become more available.

## Conclusion

We observed an increasing trend in notification rates of *S. boydii* in Australia since 2013, with most cases, whose place of acquisition was known, being acquired overseas. This emphasizes the importance of public health messaging regarding infectious diarrhea, including shigellosis, targeted to international travelers, particularly those traveling to the Indian subcontinent. Through our WGS analysis of Victorian and published isolates, we identified five phylogenetic lineages that were circulating globally over multiple decades. Our study showed high levels of AMR, with over 60% of isolates classified as MDR. Of concern, more than one-third of Australian isolates were inferred to be resistant to or have reduced susceptibility to ciprofloxacin, and although rare, resistance mechanisms to other therapeutics of azithromycin and ceftriaxone were detected. This highlights the continued need for genomic characterization of *Shigella* isolates in Australia to ensure that emerging trends in AMR patterns can be identified early to better support public health interventions and treatment recommendations. Overall, our study represents the largest analysis of *S. boydii* sequences to date and contributes to the body of evidence on the epidemiological and genomic characteristics of *S. boydii* in Australia and globally.

## Supplementary Material

S Boydii Manuscript_Supplementary Material_Table S1_updated August 2025.xlsx

S Boydii Manuscript_Supplementary Material_Aug2025_clean.docx

## Data Availability

The data underlying the epidemiological anlaysis in this study are from the National Notifiable Diseases Surveillance System. As the data was obtained from a third party, we are unable to upload this dataset. Australian shigellosis notification data, as was used in this study, can be requested from the Communicable Diseases Network Australia. Data requests can be sent to NNDSS.datarequests@health.gov.au. The genomic data in this study is available at the National Center for Biotechnology Information Short Read Archive (BioProject PRJNA857526).
