# Supplementary material for: Genomic and epidemiological characteristics of Shigella boydii in Australia, 1991–2022
Source: Pathog Glob Health. 2025 Oct 23;119(8):332–42. doi: 10.1080/20477724.2025.2573308 (PMC12897542; doi:10.1080/20477724.2025.2573308)
Supplement: S Boydii Manuscript_Supplementary Material_Aug2025_clean.docx [file YPGH_A_2573308_SM2369.docx]

## **Genomic and epidemiological characteristics of *Shigella boydii* in Australia, 1991-2022**

## **Supplementary Material**

**Table S2. Notifications and notification rate per 100,000 population of *S. boydii* by jurisdiction, Australia, 1991-2019**

| Jurisdiction | Notifications | Rate |
| --- | --- | --- |
| ACT | 1 | 0.2 |
| NSW | 77 | 1.0 |
| NT | 7 | 2.8 |
| QLD | 22 | 0.4 |
| SA | 46 | 2.6 |
| TAS | 2 | 0.4 |
| VIC | 111 | 1.7 |
| WA | 28 | 1.0 |

**Figure S1. Proportion of *S. boydii* notifications by place of acquisition, Australia, 1991-2019**

*Bar plot (by proportion) on the y axis of cases. Years shown on x axis and barplots coloured by source of acquisition.*

**
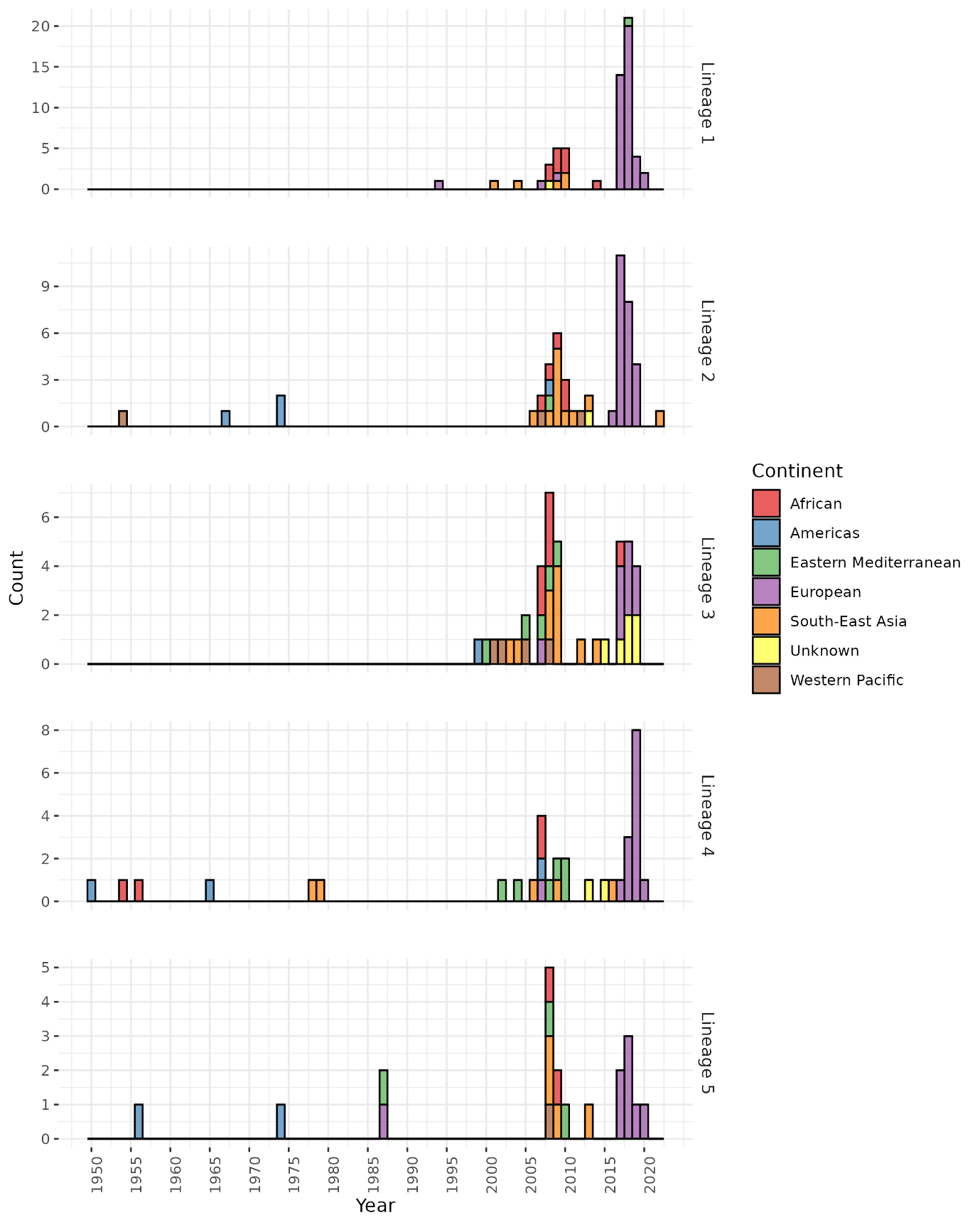
**

**Figure S2. Victorian and published *S. boydii* isolates by year and continent of collection.**

*Bar plots for the five BAPS lineages. Counts are shown on the y-axis and time (years) on the x-axis. Plots are coloured by geographical region. Note, the y-axis is not consistent for all lineages.*

**
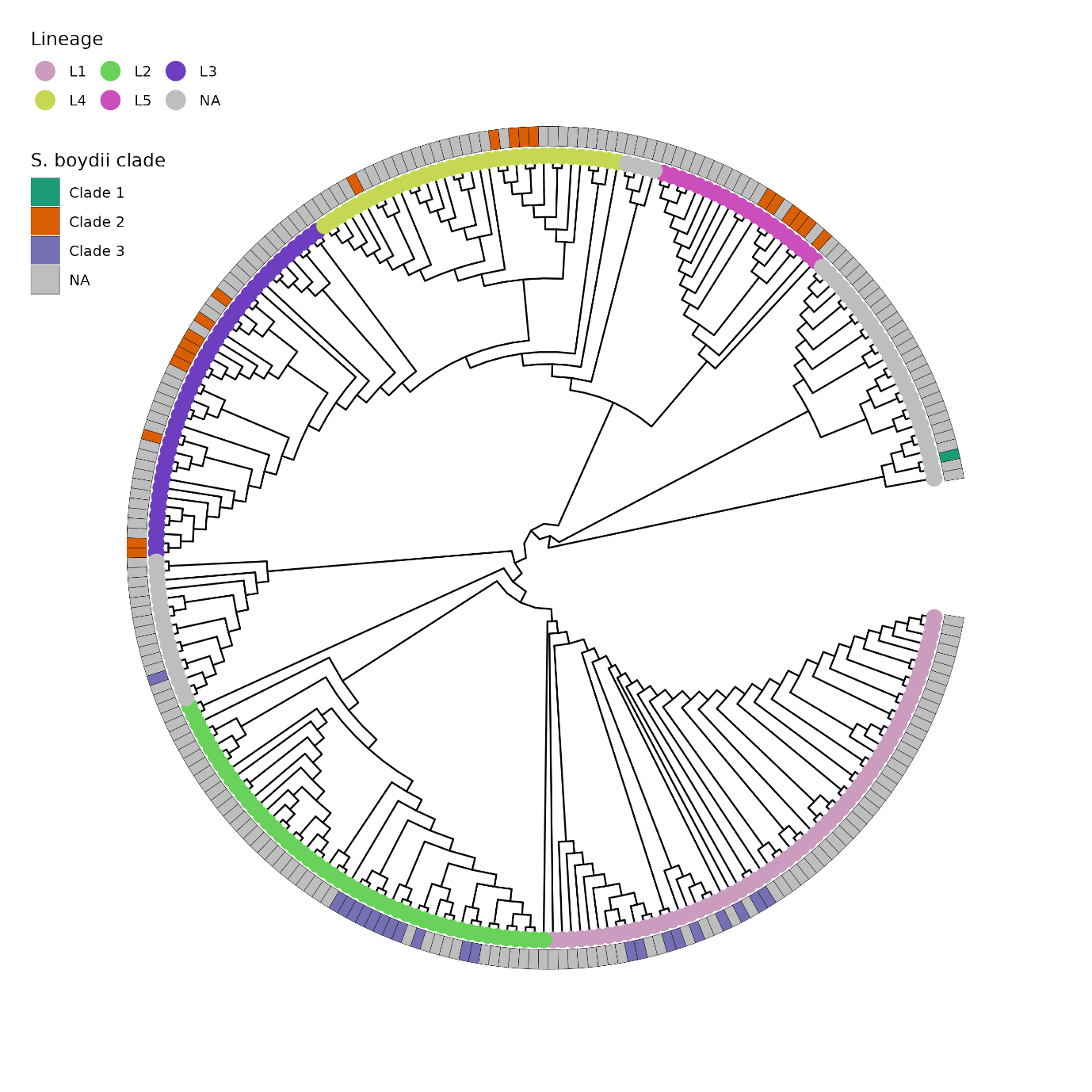
**

**Figure S3. Cladogram representing the maximum-likelihood core genome phylogeny of 250 *S. boydii* genomes and previously assigned clade designations.**
*Cladogram of 250 S. boydii genomes included in this study. Cladogram used for ease of visualisation – branch lengths do not represent evolutionary change/time. Major linages are highlighted in the phylogenetic tree tip points (L1-L5); NA represents isolates that were not classified into a major lineage. Clade names displayed are in accordance with the designations determined in Kania et al 2016.^[[1]](#footnote-1)^*


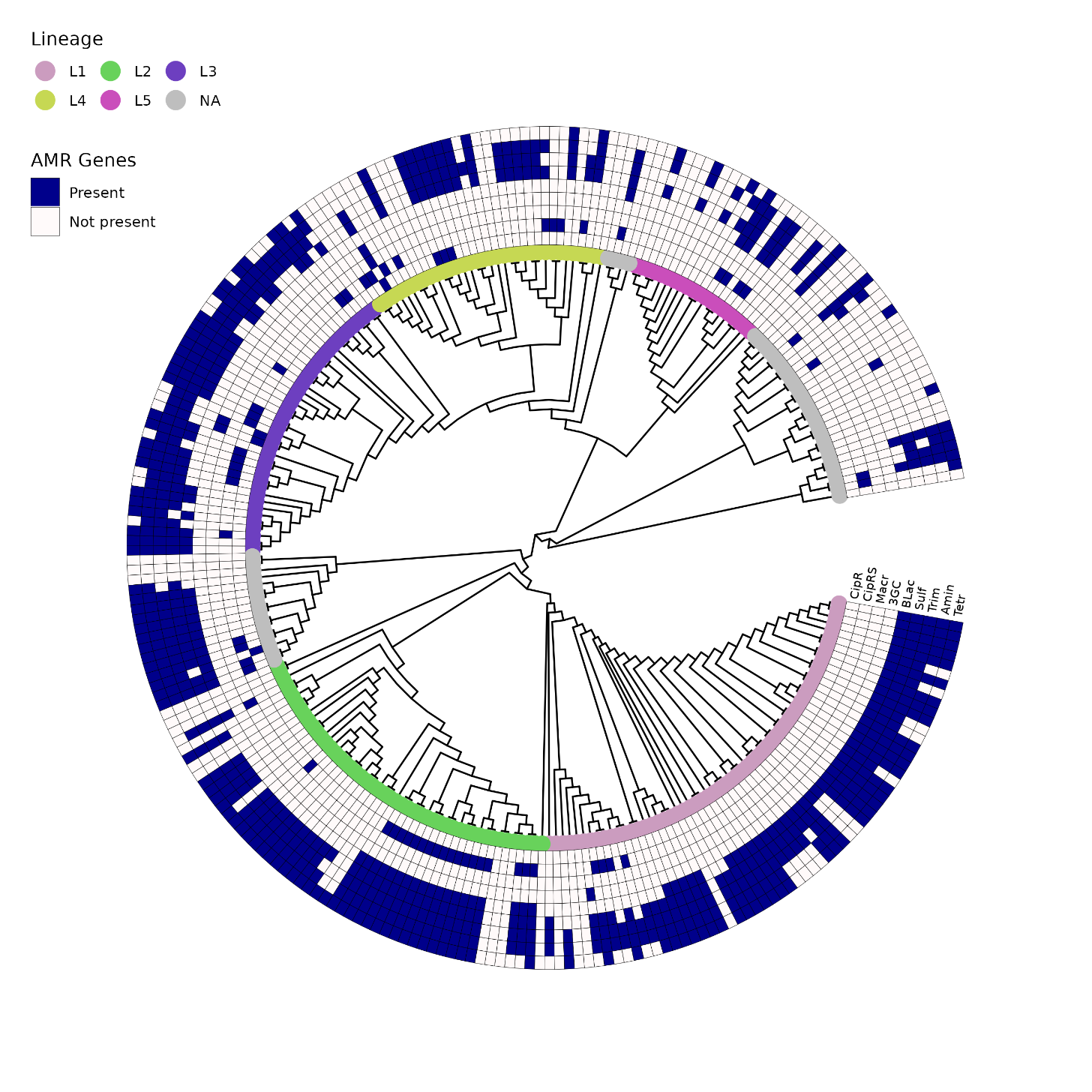


**Figure S4. Cladogram representing the maximum-likelihood core genome phylogeny of 250 *S. boydii* genomes with AMR profiles.***Cladogram of 250 S. boydii genomes included in this study. Cladogram used for ease of visualisation – branch lengths do not represent evolutionary change/time. Major linages are highlighted in the phylogenetic tree tip points (L1-L5); NA represents isolates that were not classified into a major lineage. For each isolate, the presence/absence of AMR genes are shown: Quinolone (Quin), Ciprofloxacin resistant (CipR), Ciprofloxacin reduced susceptibility (CipRS), Macrolide, 3GC, Beta lactamase: not-ESBL or carbapenemase (BLac), Sulfonamide (Sulf), Trimethoprim (Trim), Aminoglycoside (Amin), Tetracycline (Tetr).*

1. Kania, D.A., et al., Genome diversity of Shigella boydii. Pathog Dis, 2016. 74(4): p. ftw027. [↑](#footnote-ref-1)
